# Adrenomedullin alleviates mucosal injury in experimental colitis and increases claudin‐4 expression in the colonic epithelium

**DOI:** 10.1002/2211-5463.13577

**Published:** 2023-02-27

**Authors:** Makiko Kawaguchi, Hiroaki Kataoka, Takumi Kiwaki, Liang Weiting, Sayaka Nagata, Kazuo Kitamura, Tsuyoshi Fukushima

**Affiliations:** ^1^ Section of Oncopathology and Regenerative Biology, Department of Pathology University of Miyazaki Japan; ^2^ Frontier Science Research Center University of Miyazaki Japan

**Keywords:** adrenomedullin, claudin‐4, inflammatory bowel disease, mucosal regeneration

## Abstract

Adrenomedullin (AM) is a peptide with pleiotropic physiological functions that attenuates intestinal mucosal inflammation. However, the mechanism underpinning mucosal protection by AM is not fully understood, and its effect on intestinal epithelial cells remains unclear. Here, we investigated the effects of AM on junctional molecules in primary‐cultured murine intestinal epithelial cells and discovered that AM upregulates claudin‐4 expression. In a mouse model of dextran sulfate sodium‐induced colitis, AM administration also enhanced claudin‐4 expression and accelerated mucosal regeneration. Furthermore, AM reversed TNFα‐mediated downregulation of claudin‐4 and loss of cell–cell adhesion of the HCT116 human intestinal epithelial cell line *in vitro*. These results indicate that AM may enhance intestinal epithelial integrity by upregulating claudin‐4 expression.

## Graphical abstract

Adrenomedullin (AM) is a bioactive peptide with pleiotropic functions. We showed that AM promotes the regeneration of injured intestinal epithelium via the upregulation of claudin‐4 in murine DSS‐induced colitis model. Moreover, AM ameliorated the decreased expression of claudin‐4 caused by TNFα treatment in human colonic epithelial cells. These results suggest that AM may enhance intestinal epithelial integrity by upregulating claudin‐4 expression.

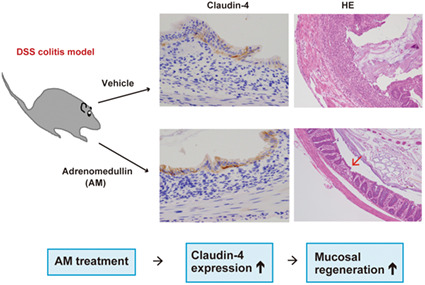

**Abbreviations**
AM: adrenomedullin
CLR: calcitonin receptor‐like receptor
DSS: dextran sulfate sodium
IBD: inflammatory bowel disease
RAMP2: Receptor activity modifying protein 2
TJ: tight junction
TNF: tumor necrosis factor

Inflammatory bowel disease (IBD), consisting of Crohn's disease and ulcerative colitis, is a chronic inflammation of the gastrointestinal tract. To date, the exact etiology of IBD remains unknown. However, multiple factors are thought to be involved in IBD pathogenesis, such as gut microbiota, genetic predisposition, and deregulation of immune response [1, 2, 3, 4]. Because there is currently no curative therapy for IBD, the development of effective therapeutics is highly advocated [5, 6].

Adrenomedullin (AM) is a bioactive peptide with pleiotropic functions, including vasodilation, angiogenesis, organ protection, mucosal healing, and immunomodulation [7, 8]. Adrenomedullin and its receptor molecules, receptor activity modifying protein 2 (RAMP2) and calcitonin receptor‐like receptor (CLR), have been discovered to be highly expressed in the gastrointestinal tract [9, 10, 11]. Therefore, many studies on the effects of AM on intestinal inflammation using animal models have been reported [12, 13, 14, 15, 16, 17, 18]. These reports suggested that AM has a beneficial effect on experimental IBD models. Moreover, it has been reported that AM may accelerate the regeneration of intestinal mucosa and may be useful for intractable IBD in clinical trials [19, 20]. In these models, AM has been reported to ameliorate colitis by inhibiting the production of inflammatory cytokines, maintaining the epithelial barrier, and stimulating angiogenesis. In a study of AM knockout mice, the lack of endogenous AM reduced the expression levels of adhesion molecules, JAM‐A and E‐cadherin. Adrenomedullin also regulates genes related to colonic epithelial regeneration such as *LGR5*, *Wnt5a*, *Egfr*, and *Erbb2* [16]. However, the effect of AM on intestinal epithelial cells remains unclear. Thus, the main aim of this study was to elucidate the direct effects of AM on intestinal epithelial cells in the process of accelerated mucosal regeneration caused by AM treatment.

## Materials and methods

### Antibodies

The following antibodies were used: anti‐β‐actin mouse monoclonal IgG (A2228; Sigma‐Aldrich, St Louis, MO, USA, 1 : 2500), anti‐Claudin‐4 rabbit monoclonal IgG (ab210796; Abcam, Cambridge, UK, 1 : 1000), anti‐ CLR rabbit polyclonal IgG (ab84467; Abcam, 1 : 1000), anti‐RAMP2 rabbit polyclonal IgG (13223‐2AP; Proteintech Japan, Tokyo, Japan, 1 : 1000), anti‐mouse CD45 rat monoclonal IgG (WTA1330; Wuxi Biosciences, San Diego, CA, USA, 1 : 50), and anti‐Villin rabbit polyclonal IgG (PA5‐22072; Thermo Fisher Scientific, Waltham, MA, USA, 1 : 50).

### Cell culture

HCT116, the human colon carcinoma cell line and used as models of human intestinal epithelial cells, was obtained from the American‐Type Culture Collection (Manassas, VA, USA). HEK293 cells that stably express functional AM receptors (RAMP2 and CLR), DSR2, had been previously established [21] and used as a control cell line. These cells were maintained in Dulbecco's modified eagle's medium (DMEM) containing 10% fetal bovine serum (Sigma) and kept at 37 °C in a humidified atmosphere containing 5% CO_2_.

For the primary culture of mouse colonic epithelial cells, large intestine tissue samples isolated from 3‐ to 4‐week‐old male mice were washed with cold phosphate‐buffered saline (PBS) and minced into 3 mm pieces. After washing again with cold PBS, the fragments were incubated for 30 min at 37 °C in Hanks' Balanced Salt Solution containing 1 mg·mL^−1^ collagenase P (Roche Diagnostics Japan K.K., Tokyo, Japan) and 2 U·mL^−1^ dispase II (Roche Diagnostics Japan K.K.), followed by centrifugation (400 *×* ***g***, 3 min). Then, the fragments were washed with PBS and seeded in L‐WRN (American‐Type Culture Collection) conditioned media [22]. After one passage, the cells were subjected to experiments.

### Measurement of the intracellular cyclic adenosine monophosphate

Cells were incubated with varying concentrations of AM for 15 min, and intracellular cAMP was measured using the Cyclic AMP ELISA Kit (Cayman Chemical, Michigan, MI, USA) according to the manufacturer's instructions [23].

### Induction of experimental colitis

All animal experiments were performed using protocols approved by the Institutional Animal Care and Use Committee of the University of Miyazaki (ethics approval number: 2018‐503). Mice were housed in specific pathogen‐free room under a 12‐h light–dark cycle at 23 ± 2 °C with free access to food and water. For colitis induction, eight‐week‐old male C57BL/6 mice were treated with dextran sulfate sodium (DSS; molecular weight 36–50 kDa; MP Biomedicals, Solon, OH, USA) in drinking water. To assess their recovery after colitis induction, mice were treated with 1% DSS for 7 days. Additionally, 80 nmol·kg^−1^ of synthetic AM in 100 μL of saline (Peptide Institute Inc., Osaka, Japan) was administered subcutaneously once a day for 7 days. The control group was administered an equal amount of saline (*n* = 14 for each group). On Day 7, the drinking water was changed to distilled water without DSS for the following 7 days. Mice were weighed every 2 days.

### Reverse transcription–polymerase chain reaction and Real‐Time RT–PCR

Total RNA was prepared with TRIzol (Life Technologies, Tokyo, Japan) followed by DNase I (Takara Bio, Shiga, Japan) treatment. For RT–PCR, 3 μg total RNA was reverse‐transcribed with a mixture of Oligo (dT) 12–18 (Life Technologies) and random primers (6‐mer; Takara Bio) using 200 units of ReverTra Ace (TOYOBO, Osaka, Japan), and 1/30 of the resulting cDNA was processed for each PCR with 0.2 μm of both forward and reverse primers and AmpliTaq Gold® PCR Master Mix (Life Technologies). The primer sequences used are described in Table 1. Real‐time RT–PCR was performed in a Thermal Cycler Dice Real‐Time System II (Takara Bio) using the SYBR Premix Ex Taq II (Takara Bio). For internal control, *β*‐actin mRNA was also measured. The primer sequences used are described in Table 1.

**Table 1:** Primer sequences for RT‐PCR and real‐time RT‐PCR.

Target	Forward	Reverse	Product size (bp)
Mouse			
*Cldn4*	5′‐TCATCGTGGCAAGCATGCTG	5′‐TAGGGCTTGTCGTTGCTACG	207
*Cldn2*	5′‐CCTCGCTGGCTTGTATTATCTCTG	5′‐GAGTAGAAGTCCCGAAGGATG	174
*Ramp2*	5′‐GCAGAGAGGATCATCTTTGAGACTC	5′‐CCTCCATACTACAAGAGTGATGAGGAAG	117
*Ramp3*	5′‐GGTCATTAGGAGCCACGTGT	5′‐GGGCTAAACAAGCCACAGCT	106
*Crlcrl*	5′‐TGCTCTGTGAAGGCATTTAC	5′‐CAGAATTGCTTGAACCTCTC	66
*Tnf*	5′‐TCGAGTGACAAGCCTGTAGC	5′‐GGAGGTTGACTTTCTCCTGG	255
*Villin*	5′‐TTATGAGCCCGAAAGTGGACG	5′‐CAAGGCCCTAGTGAAGTCTTC	173
*Epcam*	5′‐CTTCAAGAGGCGTTCACATC	5′‐ACATCAGCTATGTCCACGTC	140
*Actb*	5′‐TGACAGGATGCAGAAGGAGA	5′‐GCTGGAAGGTGGACAGTGAG	131
Human			
*CLDN4*	5′‐TATGGATGAACTGCGTGGTG	5′‐CACGATGATGCTGATGATGAC	122
*CLDN2*	5′‐CTCCCTGGCCTGCATTATCTC	5′‐ACCTGCTACCGCCACTCTGT	91
*RAMP2*	5′‐GCAGAGAGGATCATCTTTGAGACTC	5′‐CCTCCATACTACAAGAGTGATGAGGAAG	156
*RAMP3*	5′‐CCGAGTTCATCGTGTACTATGAGAG	5′‐CTGTGGATGCCGGTGATGAAGC	115
*CALCRL*	5′‐CTGTACATGAAAGCTGTGAGAGCTACT	5′‐TGGAAGTGCATAAGGATGTGCATGATG	140
*ACTB*	5′‐ATTGCCGACAGGATGCAGA	5′‐GAGTACTTGCGCTCAGGAGGA	89

### Stimulation of cells with TNFα

To mimic intestinal inflammation *in vitro*, HCT116 cells were incubated with 100 ng·mL^−1^ TNFα (R&D systems, Minneapolis, MN, USA) for 18 h. At the same time, 10 or 100 nm AM was added to examine its effect on inflammation.

### Polymerase chain reaction array assay for cell adhesion molecules

Mouse intestinal epithelial cells were cultured with or without 10 nm synthetic AM for 24 h. Total RNA was extracted from the cells using Trizol. mRNA levels of the cell adhesion molecules were assessed by real‐time PCR with cell adhesion molecules (Primer Array® Cell Adhesion Molecules (Mouse), TaKaRa Bio) and SYBR Premix Ex Taq II (TaKaRa Bio). The experiments were repeated three times, and changes in more than 1.5 fold with a coefficient of variation of less than 25% in the three independent experiments were considered significant.

### Histological analyses

Intestinal tissue samples were fixed overnight in 4% paraformaldehyde in PBS and then dehydrated and embedded in paraffin. Furthermore, 4‐μm‐thick sections were prepared and stained with hematoxylin and eosin. For immunohistochemistry, the staining was performed on a Leica Bond Max III automated immunostainer (Leica Biosystems, Tokyo, Japan) according to the manufacturer's instructions. To quantify CD45‐positive cells, stained sections were selected and photographed at 200× magnification. Then, two independent investigators counted the CD45‐positive cells, and the mean number per field was calculated.

### Immunoblot analysis

Cellular proteins were extracted with RIPA buffer (Nacalai Tesque, Inc., Kyoto, Japan) supplemented with protease inhibitor cocktail (Sigma), 100‐mmol·L^−1^ NaF, and 1 mol·L^−1^ Na_3_VO_4_, centrifuged at 15 000 × ***g*** for 15 min, and supernatants were collected. Equal amounts of total proteins were separated by sodium dodecyl sulfate‐polyacrylamide gel electrophoresis under reducing conditions using 4–12% gradient gels and transferred onto an Immobilon membrane (Millipore, Bedford, MA, USA). After blocking using 5% nonfat milk in Tris‐buffered saline with 0.1% Tween 20 (TBS‐T), membranes were incubated overnight at 4 °C, with a primary antibody described above. Membranes were washed with TBS‐T and incubated with horseradish peroxidase‐conjugated secondary antibody, goat anti‐mouse IgG (#1706516; Bio‐Rad Laboratories Inc., Hercules, CA, USA, 1 : 5000), or swine anti‐rabbit IgG (P0217; Dako, Glostrup, Denmark, 1 : 5000) diluted in TBS‐T with 1% bovine serum albumin for 1 h at room temperature. The labeled proteins were visualized with a chemiluminescence reagent (PerkinElmer Life Science, Boston, MA, USA).

### Measurement of transepithelial electrical resistance

Measurement of transepithelial electrical resistance (TEER) was taken as described previously [24, 25, 26]. Briefly, HCT116 cells (5 × 10^4^) were seeded into the 24‐well insert of Transwell (Corning Costar, New York, NY, USA) and allowed to reach confluence. After treatment with TNFα (100 ng·mL^−1^) for 18 h with (100 nm) or without AM, TEER were measured with Millicell‐ERS Electrical Resistance System (Millipore). A culture medium without cells was used as the negative control.

### Measurement of intestinal permeability

To quantify the paracellular permeability *in vitro*, HCT116 cells (5 × 10^4^) were seeded into the 24‐well insert of Transwell (Corning) and allowed to reach confluence. When cells were reached confluence, the medium was changed with serum‐free DMEM and cells were treated with TNFα (100 ng·mL^−1^) for 18 h with (100 nm) or without AM. Subsequently, 1.0 mg·mL^−1^ of fluorescein isothiocyanate‐dextran (FD‐4; Sigma) was added to the apical compartment of transwell and incubate for 2 h. Then, the basel culture medium was collected, and the fluorescence intensity of FD‐4 was detected using fluorometer (DTX800 Multimode Detector; Beckman Coulter, Fullerton, CA, USA) with excitation an emission wavelengths of 485 and 535 nm, respectively. FITC‐dextran concentrations were determined from standard curves generated by serial dilution of FITC‐dextran.

### Statistical analysis

Comparison between two unpaired groups was made with repeated measure analysis of variance, Student's *t*‐test or Mann–Whitney *U*‐tests using stat view 5.0 (SAS Institute Inc., Cary, NC, USA). For the primer array experiment, paired *t*‐test was performed. The threshold for statistical significance was *P* < 0.05.

## Results

### Adrenomedullin receptor is expressed in colonic epithelial cells

**Fig. 1:**
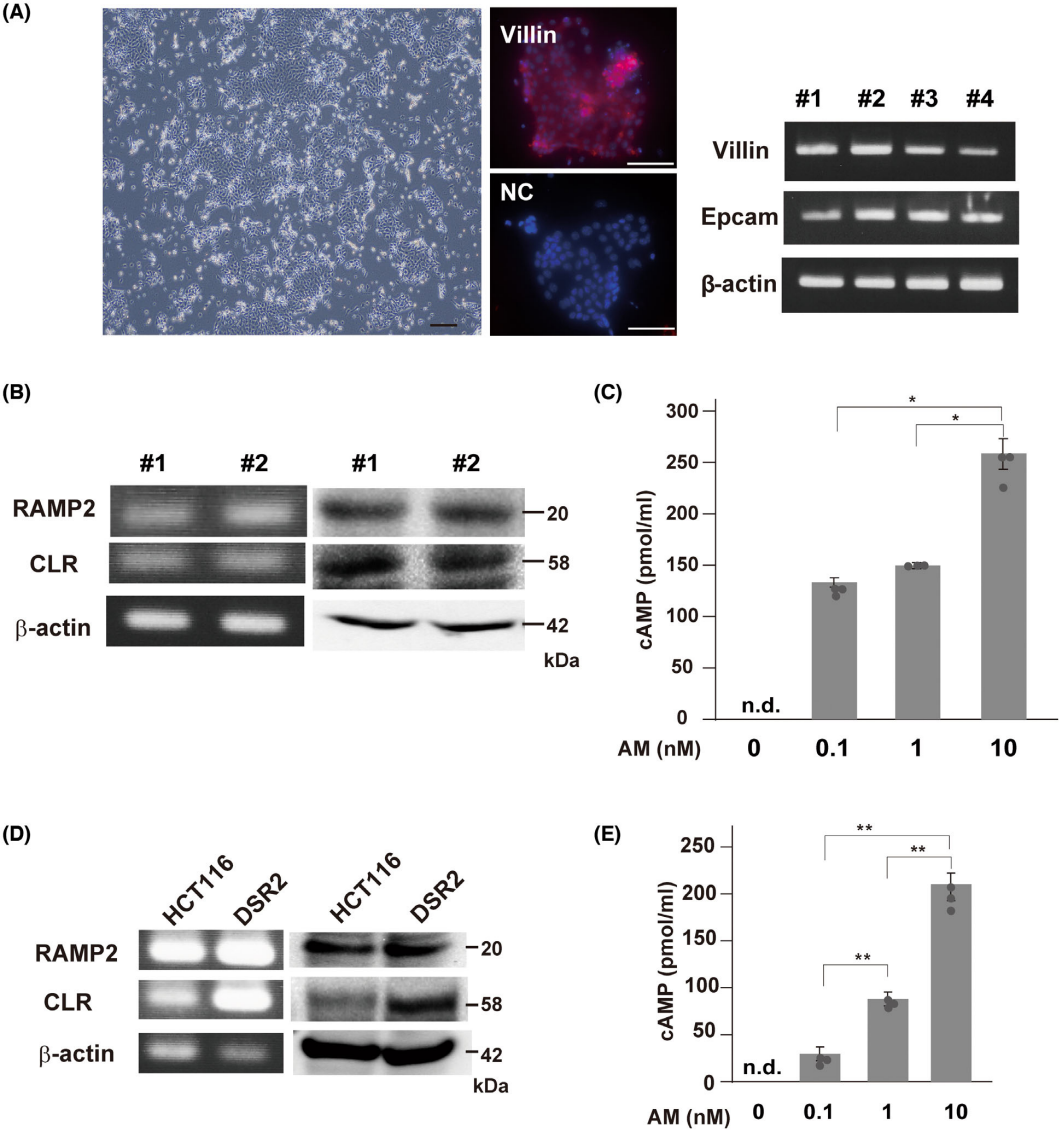
Primary culture of murine colonic epithelial cells. (A) Representative culture morphology of cultured colonic epithelial cells and immunofluorescence staining of Villin (left panel). Bars, 100 μm. RT–PCR for the Villin and Epcam genes (right panel). Data from four independent primary cultures are shown. (B) RT–PCR (left panel) and immunoblot analysis (right panel) of AM receptor molecules, RAMP2 and CLR, in the primary‐cultured murine colonic epithelial cells. Data from two independent primary cultures are shown. (C) AM‐mediated intracellular accumulation of cAMP in murine colonic epithelial cells. Data are presented as the means ± standard deviation (SD) of three independent experiments. n.d, not detectable. Circles represent the value of each case. *P < 0.05; Student's *t*‐test. (D) Representative data of RT–PCR (left panel) and immunoblot (right panel) of RAMP2 and CLR in HCT116 and DSR2 (HEK293 cells that stably express functional RAMP2 and CLR) cells. (E) Intracellular cAMP accumulation by AM in HCT116. Data are presented as the means ± SD of three independent experiments. Circles represent the value of each case.**P < 0.0001; Student's *t*‐test.

To analyze the roles of AM in colonic epithelial cells, we performed a primary culture of murine colonic epithelial cells. We confirmed the expression of the Villin protein by immunofluorescence and *Vil1* and *Epcam* by RT–PCR to validate that the cells are epithelial cells (Fig. 1A). First, we examined the expression of AM receptor molecules, RAMP2 and CLR, in primary‐cultured murine colonic epithelial cells. These cells expressed RAMP2 and CLR in both mRNA and protein levels. However, another AM receptor molecule, RAMP3, is not expressed in murine colonic epithelial cells (Fig. 1B and Fig. S1A). Next, we checked whether the AM receptor in them is functional upon AM stimulation. Adrenomedullin treatment dose‐dependently increased intracellular cAMP levels (Fig. 1C). We also investigated the effect of AM on the human colon epithelial cell line, HCT116. As shown in Fig. 1D, HCT116 expressed AM receptor molecules, RAMP2 and CLR, whereas RAMP3 is not expressed (Fig. S1A) and AM treatment induced intracellular accumulation of cAMP (Fig. 1E).

### Adrenomedullin stimulates claudin‐4 expression in colonic epithelial cells

**Fig. 2:**
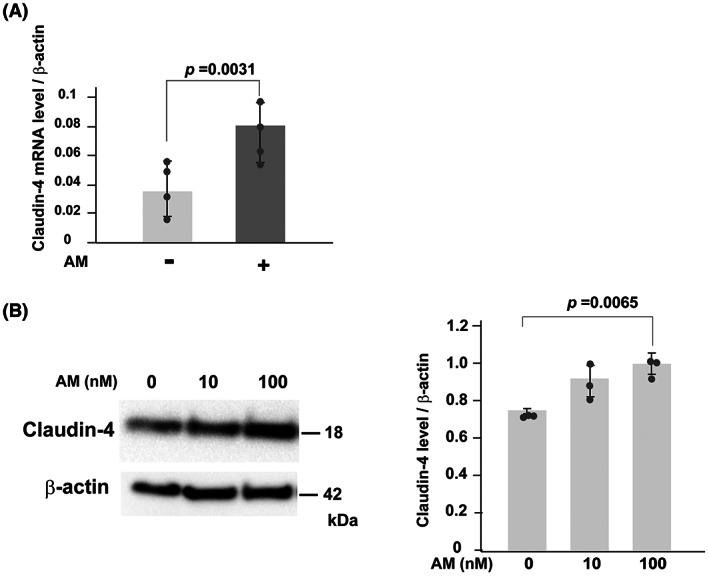
Effects of AM (10 nm) on the expression of claudin‐4 in murine primary‐cultured colonic epithelial cells. (A) Quantitative RT–PCR for the claudin‐4 mRNA in mouse colonic epithelial cells treated with or without AM treatment (10 nm). Data are means ± SD of four independent experiments. Circles represent the value of each case. *P*‐value; Mann–Whitney *U*‐test. (B) Immunoblot analysis of claudin‐4 proteins in mouse colonic epithelial cells treated with or without AM treatment (left panel). Quantitative data of claudin‐4 protein levels are indicated in the right panel. Data are means ± SD of three independent experiments. Circles represent the value of each case. *P*‐value; Student's *t*‐test.

The expression of intercellular adhesion molecules is essential for maintaining intestinal epithelial integrity. Thus, we analyzed whether AM alters the expression of cell adhesion molecules in murine colonic epithelial cells. The expression of several genes associated with cell adhesion was upregulated after AM treatment (Table 2). Among them, the expression of *Cldn4*, encoding claudin‐4, was consistently upregulated in three independent experiments (Table 2). The increased expression levels of claudin‐4 mRNA and protein in response to AM treatment were further confirmed by RT–PCR (Fig. 2A and Fig. S1B) and immunoblotting (Fig. 2B), respectively.

**Table 2:** Effect of AM treatment on gene expressions of cell adhesion molecules (Primer Array).

Gene name	Ex1^a^	Ex2^a^	Ex3^a^	*P* value*
*Itga6*	1.163	1.003	1.039	0.2939
*L1cam*	1.238	1.297	1.653	0.0925
*Ocln*	1.264	1.809	1.886	0.0793
*Cdh1*	0.9444	0.8039	0.7145	0.1159
*Cldn3*	0.9845	0.7983	1.510	0.6919
*Cldn4*	1.599	2.370	2.342	**0.0485**
*Itgb1*	1.147	1.380	1.532	0.0876
*Ptprf*	1.212	1.371	2.354	0.2123
*Sdc1*	1.281	1.279	3.127	0.283
*Sdc4*	0.9444	1.333	1.619	0.266
*Cldn7*	1.123	1.025	1.619	0.2989
*Cldn8*	0.9444	2.630	2.437	0.2001
*Cldn1*	0.3386	0.976	1.821	0.9258
*F11r*	1.412	1.380	1.723	**0.0439**

^a^ Values are fold change compared to the untreated group.

* Paired *t*‐test. Statistical significant values (*p* < 0.05) are indicated in bold.

### Adrenomedullin administration upregulates claudin‐4 expression and alleviates the weight loss accompanied with dextran sulfate sodium‐induced experimental colitis

**Fig. 3:**
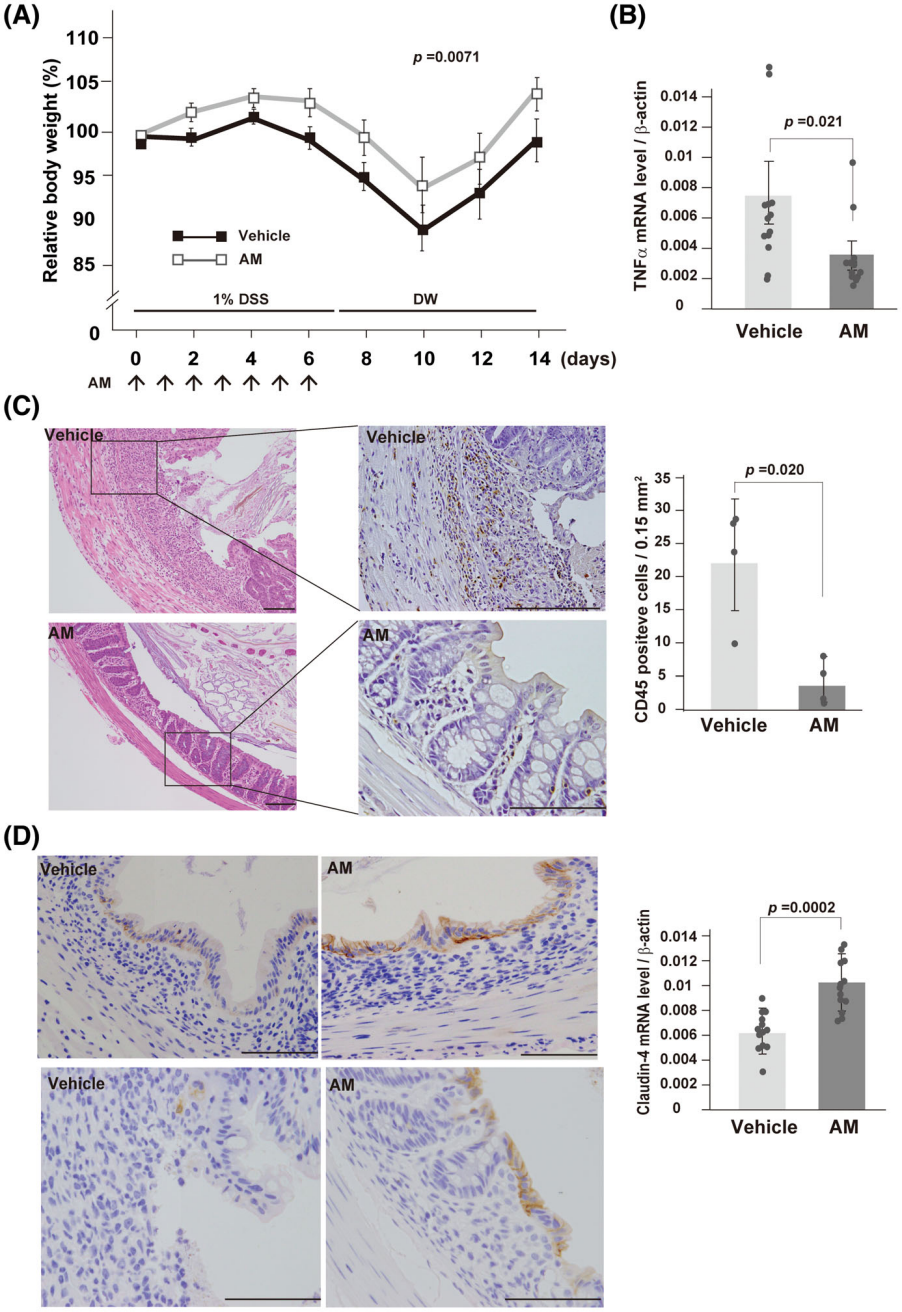
Effects of AM on mouse DSS‐induced colitis. (A) Body weight changes for the AM‐treated group (AM, *n* = 14) and vehicle (saline)‐treated control group (Vehicle, *n* = 14). Arrows indicate treatment points. *P*‐value; two‐way repeated‐measures ANOVA. (B) Quantitative RT–PCR for TNFα mRNA. Error bars, standard deviation (SD; *n* = 13 in each group). Circles represent the value of each case. *P*‐value; Mann–Whitney *U*‐test. (C) Representative images of histology by hematoxylin and eosin staining, CD45 immunohistochemistry (left panel) and quantitation of CD45‐positive cells (right panel) of the large intestine on the 14th day (7 days after stopping DSS treatment). The black boxes that overlay the left panels (HE stains) indicate the region shown in the right panels (CD45 immunostains). Values are the means ± SD (*n* = 4 in each group). Circles represent the value of each case. *P*‐value; Mann–Whitney *U*‐test. (D) Representative images of low‐magnification micrographs from non‐ulcer regions (upper micrographs) and high‐magnification from ulcer regions (lower micrographs) following claudin‐4 immunohistochemistry. Quantitative RT–PCR of the expression levels of claudin‐4 mRNA is shown on the right. Data are presented as the means ± SD (*n* = 13 in each group). Circles represent the value of each case. *P*‐value; Mann–Whitney *U*‐test. Bars, 100 μm.

To investigate whether the expression of claudin‐4 was upregulated after AM treatment *in vivo*, we used an experimental colitis model induced by oral administration of 1% DSS. Consistent with previous studies [17, 18], AM treatment alleviates the severity of DSS‐induced colitis. Mice treated with 80 nmol·kg^−1^ AM had significantly less body weight loss than control mice (Fig. 3A). Furthermore, AM treatment exerted inhibitory effects on DSS‐induced inflammatory changes as judged by the reduced expression of TNFα and immunoreactivity of CD45 (Fig. 3B,C). Consequently, the regeneration of intestinal mucosa was accelerated by the AM treatment (Fig. 3C). In accordance with the above *in vitro* results, claudin‐4 expression was also increased in the AM‐treated group (Fig. 3D). The enhanced claudin‐4 expression was more evident in the regenerative epithelium (Fig. 3D and Fig. S1C).

### Adrenomedullin ameliorates claudin‐4 expression in TNFα‐treated HCT116 cells

**Fig. 4:**
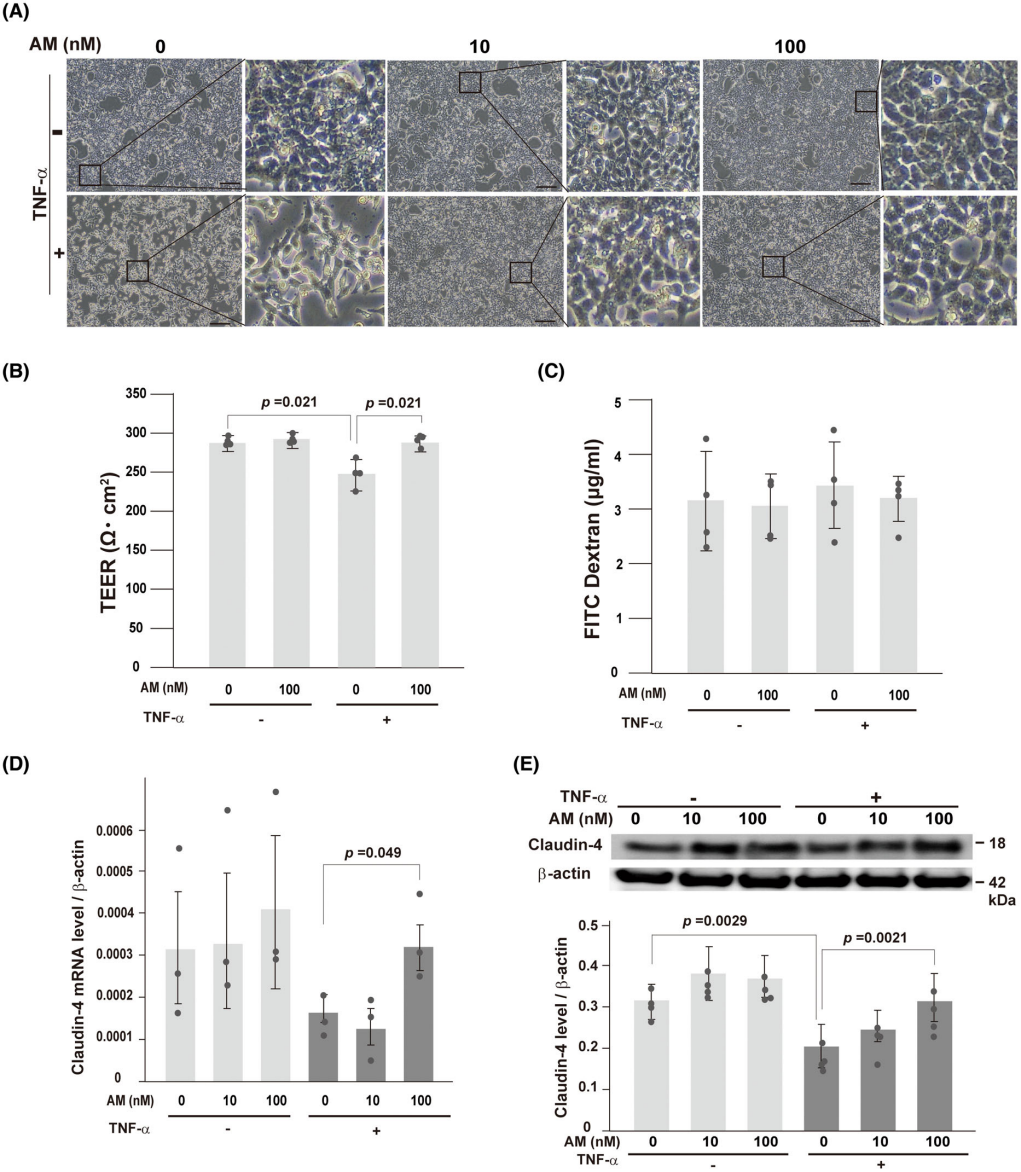
Effects of AM on the expression of claudin‐4 in human colonic epithelial cell line, HCT116. (A) Culture morphologies of HCT116 cells after treatment with TNFα (100 ng·mL^−1^) for 18 h with or without AM. Bars, 50 μm. (B) Transepithelial electrical resistance (TEER) values of HCT116 cells after treatment with TNFα (100 ng·mL^−1^) for 18 h with (100 nm) or without AM. Values are the means ± SD of three independent experiments. Circles represent the value of each case. *P*‐value; Mann–Whitney *U*‐test. (C) Concentration of fluorescein isothiocyanate‐dextran (FD‐4) of HCT116 cells after treatment with TNFα (100 ng·mL^−1^) for 18 h with (100 nm) or without AM. Values are the means ± SD of three independent experiments. Circles represent the value of each case. (D) Quantitative RT–PCR for the claudin‐4 mRNA. Data are presented as the means ± SD of three independent experiments. Circles represent the value of each case. *P*‐value; Student's *t*‐test. (E) Immunoblot analysis for claudin‐4 in HCT116 cells after TNFα treatment with or without AM (upper panel). Quantitative data of claudin‐4 protein levels are indicated in the lower panel. Data are presented as the means ± SD of four independent experiments. Circles represent the value of each case. *P*‐value; Mann–Whitney *U*‐test.

Next, we investigated the effect of AM on claudin‐4 expression in the TNFα‐treated human colonic epithelial cell line, HCT116, which mimics the inflammatory condition of epithelial cells in colitis. The cells were treated with 100 ng·mL^−1^ of TNFα with or without AM. As shown in Fig. 4A, TNFα reduced cell–cell adhesion, and this effect was alleviated by AM treatment. We also investigated the effects of AM on the intestinal barrier functions. The TEER values was lower in TNFα treated HCT116 cells in a statistically significant level, whereas AM treatment ameliorated the TEER values (Fig. 4B). The concentration of FD‐4 tended to increase after TNFα treatment and decreased after AM treatment. However, the differences were not significant (Fig. 4C). The expression of claudin‐4 was also decreased by TNFα treatment, and AM significantly ameliorated the expression of claudin‐4 at both mRNA and protein levels (Fig. 4D,E).

## Discussion

Adrenomedullin is a biologically active peptide isolated from human pheochromocytoma tissue [7] and known to have various physiological functions, such as angiogenesis, organ protection, and anti‐inflammatory activity [8]. Adrenomedullin is widely expressed by the gastrointestinal epithelium and plays protective roles against gastrointestinal diseases, such as gastric ulcer or IBD, in animal models [18, 27, 28]. The clinical trials of AM have been conducted as a novel therapeutic agent for IBD in humans [8, 19, 20]. In a double‐blind, randomized trial, complete remission was observed at 8 weeks in patients with steroid‐resistant ulcerative colitis receiving a high dose of AM [19]. However, it is still unclear whether AM had a direct effect on the intestinal epithelial cells. Herein, we revealed that AM promoted the expression of claudin‐4, an epithelial tight junction (TJ) protein, in primary‐cultured murine colonic epithelial cells *in vitro* and in a murine DSS‐induced experimental colitis model *in vivo*. Moreover, AM alleviated the loss of cell–cell adhesion and decreased claudin‐4 expression caused by TNFα, a proinflammatory cytokine involved in IBD pathogenesis, in the human colonic epithelial cell line, HCT116. These results suggest that AM has a direct effect on the intestinal epithelial cells to maintain epithelial integrity, thereby promoting the regeneration of intestinal mucosa after inflammation.

Claudins are transmembrane proteins and one of the TJ constituent proteins. TJ proteins constitute the intestinal epithelial barrier that regulates paracellular permeability [29]. It is well‐known that claudin‐1, 3–6, 8, 12, 18 and 19 strengthen the epithelial barrier, whereas claudin‐2 and 15 weaken it [30, 31]. In IBD, the reduction in TJ protein has been considered a trigger in intestinal epithelial barrier dysfuntion [32]. In this study, we examined the expression of claudin‐2, a leaky claudin and is increased in patients with IBD, in mouse DSS colitis model and in HCT116 cells; however, there was no significant difference in the expression of claudin‐2 after treatment with AM (Fig. S2). It has been reported that claudin‐4 staining is strong in the normal colonic epithelium with reduced immunoreactivity in the IBD surface epithelium, especially in ulcerative coliits [33].

Moreover, upregulation of claudin‐4 expression increased transepithelial resistance [34]. In DSS‐induced colitis mice, the mice showed abnormal colonic structure, barrier dysfunction, and decreased expression of claudin‐4 [26, 35]. Similarly, in Caco‐2 human colon epithelial cells, TNFα treatment decreased claudin‐4 expression accompanied by a decreased transepithelial resistance level [26, 35]. These observations indicate a crucial role of claudin‐4 expression in the epithelial barrier function of the intestine. Consequently, molecules that enhance claudin‐4 expression may represent a potential therapeutic option for the protection of the intestinal epithelium in ulcerative colitis. Studies have also demonstrated that somatostatin, a neuroendocrine peptide, restored intestinal epithelial barrier function by upregulating claudin‐4 expression in mice with DSS‐induced colitis [26]. Peptide growth factors, such as transforming growth factor (TGF)‐β [36, 37] and epidermal growth factor (EGF) [38, 39, 40, 41, 42], also upregulate claudin‐4 expression. This study revealed that AM is a novel addition to the list of epithelial barrier‐protecting peptides, providing a rationale for AM in treating IBD.

There are a couple of limitations to this study. First, the molecular mechanism by which AM enhances claudin‐4 expression remains unexplored. The mucosa‐protecting action of somatostatin is reportedly mediated by the suppression of NF‐ĸB‐MLCK‐MLC signaling [26, 35]. Caudal‐Type homeobox 2 (Cdx2), a member of the caudal‐related homeobox transcription factor gene family, has also been reported to facilitate claudin‐4 expression in gastric carcinoma cells [43]. Second, the expression of claudin‐4 and other TJ proteins in human intestinal mucosa tissue samples from patients with IBD with or without AM treatment need to be examined. Future studies should look forward in addressing these limitations.

In summary, the results of this study suggest that AM promotes the regeneration of injured intestinal epithelium via the upregulation of claudin‐4, and AM significantly ameliorated the decreased expression of claudin‐4 caused by TNFα treatment, providing an implication of AM for IBD cases refractory to the current anti‐inflammatory therapies.

## Conflict of interest

This study was (partly) supported by a collaboration research fund from Himuka AM Pharma Corp., Japan. SN and KK have stock in the company.

## Author contributions

MK, HK, and TF designed the experimental procedures. MK, LW, and TK performed the experiments. MK, HK, and TF analyzed the data. SN and KK provided the materials and necessary experimental techniques. MK, HK, and TF wrote the manuscript.

## Supporting information


**Fig. S1.** RT‐PCR analysis of RAMP3 in colonic epithelial cells and expression of claudin‐4 mRNA and protein in mouse models.

**Fig. S2.** Quantitative RT‐PCR analysis of claudin‐2 in mouse models and HCT116 cells.

## Data Availability

The data that support the findings of this study are available from the corresponding author upon reasonable request.
