# Supplementary figures and images for: Adrenomedullin alleviates mucosal injury in experimental colitis and increases claudin‐4 expression in the colonic epithelium
Source: FEBS Open Bio. 2023 Feb 27;13(4):713–23. doi: 10.1002/2211-5463.13577 (PMC10068314; doi:10.1002/2211-5463.13577)

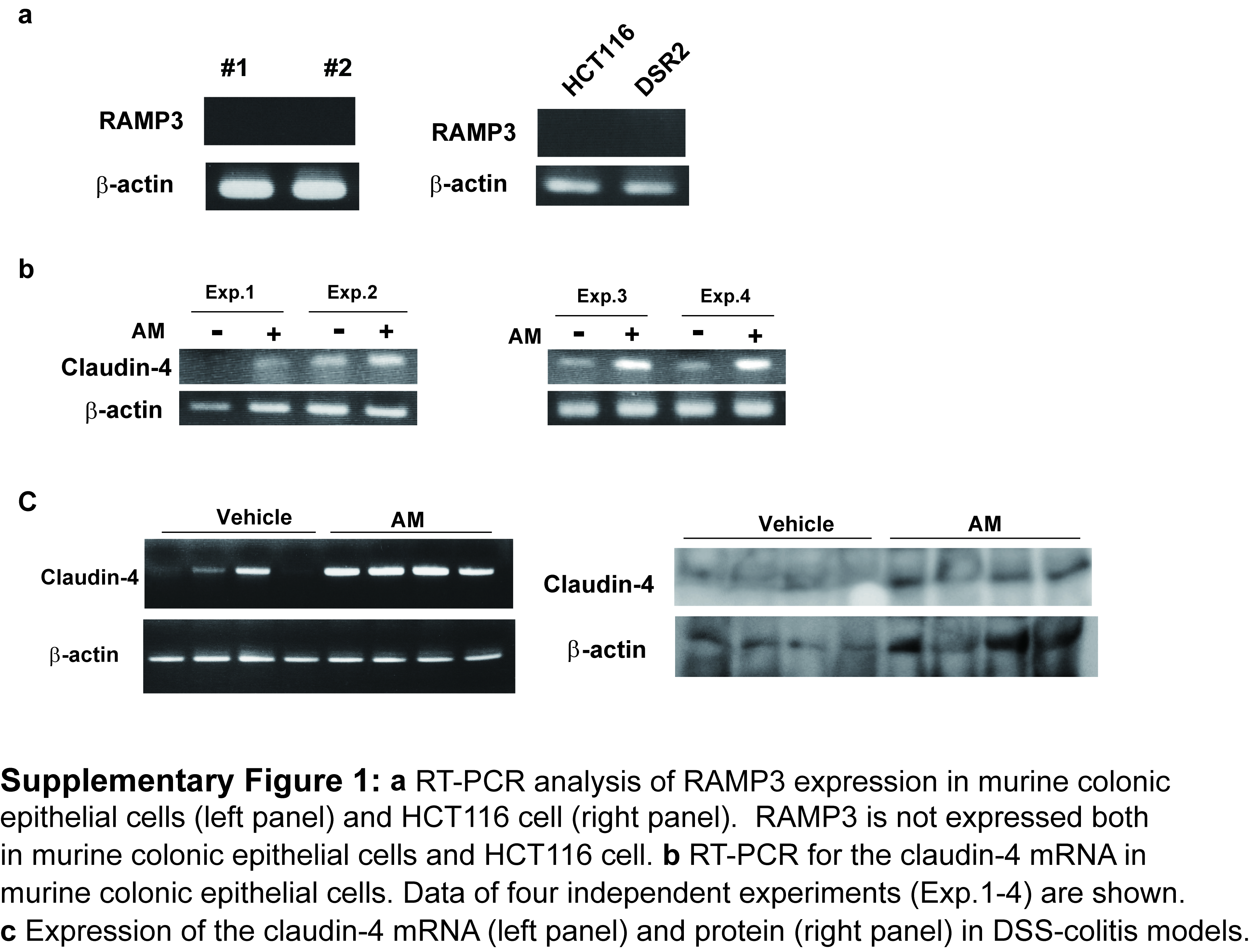

Supplement: Supplementary file 1 — Fig. S1. RT‐PCR analysis of RAMP3 in colonic epithelial cells and expression of claudin‐4 mRNA and protein in mouse models. [file FEB4-13-713-s001.tif]

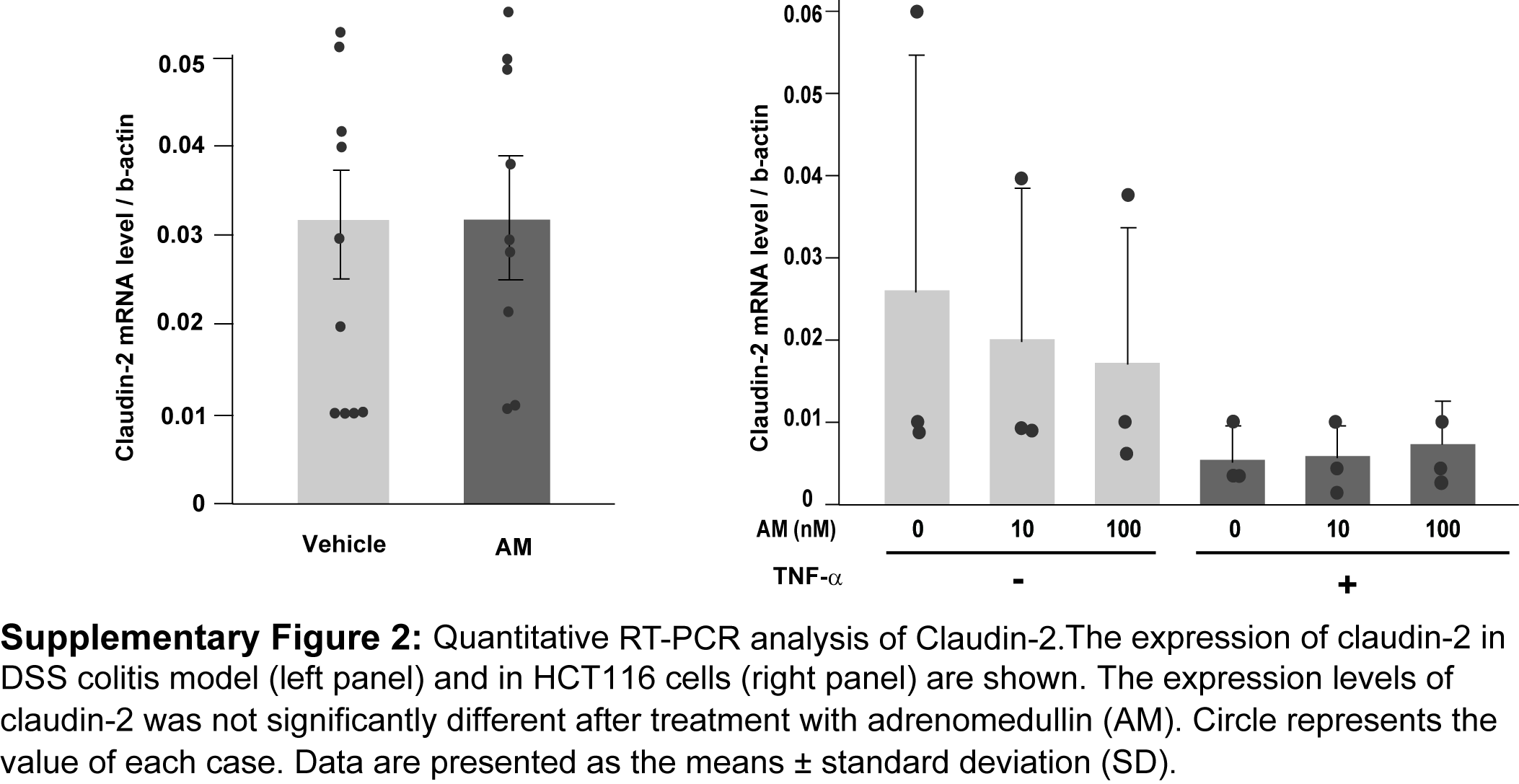

Supplement: Supplementary file 2 — Fig. S2. Quantitative RT‐PCR analysis of claudin‐2 in mouse models and HCT116 cells. [file FEB4-13-713-s002.tif]
